# Impact of Multiple Ecological Stressors on a Sub-Arctic Ecosystem: No Interaction Between Extreme Winter Warming Events, Nitrogen Addition and Grazing

**DOI:** 10.3389/fpls.2018.01787

**Published:** 2018-11-30

**Authors:** Stef Bokhorst, Matty P. Berg, Guro K. Edvinsen, Jacintha Ellers, Amber Heitman, Laura Jaakola, Hanne K. Mæhre, Gareth K. Phoenix, Hans Tømmervik, Jarle W. Bjerke

**Affiliations:** ^1^Norwegian Institute for Nature Research, FRAM – High North Research Centre for Climate and the Environment, Tromsø, Norway; ^2^Department of Ecological Science, Vrije Universiteit Amsterdam, Amsterdam, Netherlands; ^3^Community and Conservation Ecology Group, Groningen Institute for Evolutionary Life Sciences, Groningen, Netherlands; ^4^Faculty of Biosciences, Fisheries and Economics, Norwegian College of Fishery Science, UiT The Arctic University of Norway, Tromsø, Norway; ^5^Norwegian Institute of Bioeconomy Research, Ås, Norway; ^6^Climate Laboratory Holt, Department of Arctic and Marine Biology, UiT The Arctic University of Norway, Tromsø, Norway; ^7^Department of Animal and Plant Sciences, University of Sheffield, Sheffield, United Kingdom

**Keywords:** cryptogam, CO_2_ fluxes, fatty acids, frost, geometrid moth, herbivory, multiple stress, snow

## Abstract

Climate change is one of many ongoing human-induced environmental changes, but few studies consider interactive effects between multiple anthropogenic disturbances. In coastal sub-arctic heathland, we quantified the impact of a factorial design simulating extreme winter warming (WW) events (7 days at 6–7°C) combined with episodic summer nitrogen (+N) depositions (5 kg N ha^-1^) on plant winter physiology, plant community composition and ecosystem CO_2_ fluxes of an *Empetrum nigrum* dominated heathland during 3 consecutive years in northern Norway. We expected that the +N would exacerbate any stress effects caused by the WW treatment. During WW events, ecosystem respiration doubled, leaf respiration declined (-58%), efficiency of Photosystem II (Fv/Fm) increased (between 26 and 88%), while cell membrane fatty acids showed strong compositional changes as a result of the warming and freezing. In particular, longer fatty acid chains increased as a result of WW events, and eicosadienoic acid (C20:2) was lower when plants were exposed to the combination of WW and +N. A larval outbreak of geometrid moths (*Epirrita autumnata* and *Operophtera brumata*) following the first WW led to a near-complete leaf defoliation of the dominant dwarf shrubs *E. nigrum* (-87%) and *Vaccinium myrtillus* (-81%) across all experimental plots. Leaf emergence timing, plant biomass or composition, NDVI and growing season ecosystem CO_2_ fluxes were unresponsive to the WW and +N treatments. The limited plant community response reflected the relative mild winter freezing temperatures (-6.6°C to -11.8°C) recorded after the WW events, and that the grazing pressure probably overshadowed any potential treatment effects. The grazing pressure and WW both induce damage to the evergreen shrubs and their combination should therefore be even stronger. In addition, +N could have exacerbated the impact of both extreme events, but the ecosystem responses did not support this. Therefore, our results indicate that these sub-arctic *Empetrum*-dominated ecosystems are highly resilient and that their responses may be limited to the event with the strongest impact.

## Introduction

The Arctic is experiencing extreme weather events more frequently due to climate change, causing high mortality rates among species when events surpass survival thresholds (Liston and Hiemstra, 2011; Kivinen et al., 2017). Especially during the winter period more winter extreme events are expected, such as rain on snow, unseasonal warm periods, ground ice formation and loss of snow cover (Vikhamar-Schuler et al., 2016). Abrupt changes in winter snow cover and depth following mid-winter thaw events (e.g., due to temperature rise from -20°C to +5°C in just 24 h) negatively affect plant survival, detectable across landscapes through remote sensing, because of reduced snow insulation against low temperature extremes (Bokhorst et al., 2009, 2018). In addition, loss of snow cover during a midwinter melt can induce plant physiological activity (i.e., respiration and fluorescence), disrupting winter dormancy of plants, and often resulting in high mortality due to drought and frost stress (Ögren, 1996; Schaberg et al., 1996; Bokhorst et al., 2009, 2010). These extreme winter warming events (WW) occur against a background of gradually increasing temperature and extreme events such as intense herbivory by seasonal population explosions of defoliating insects (Jepsen et al., 2008) and nitrogen (N) deposition events originating from the industrialized regions further south (Karlsson et al., 2013). A combination of stressors may enhance the individual effects (Crain et al., 2008; Phoenix et al., 2012; Liess et al., 2016) and therefore have large impacts on plant development, such as phenology (Bokhorst et al., 2008), but also the plant community composition and its role in the sub-arctic terrestrial carbon budget. As such, a combination of these stressors may, through changing the dominance of plant functional types, affect the carbon balance of these sub-arctic ecosystems with potential feedbacks to greenhouse gas induced climate warming (De Deyn et al., 2008). Moreover, these community responses may differ greatly from summer warming responses and we therefore, need to understand the species and community responses for future sub-arctic vegetation predictions.

Recent experimental field studies of WW events in the sub-arctic have shown great vulnerability of evergreen dwarf shrubs to such events while deciduous plants and cryptogams are less affected (Bjerke et al., 2011, 2017a; Bokhorst et al., 2015), which may govern future sub-arctic vegetation changes (Bokhorst et al., 2015). Differences in vulnerability between plant types are partly determined by differences in exposure of overwintering tissue to freezing, and in part by physiological adaptations, such as winter dormancy and changes in the membrane fatty acid composition (Bokhorst et al., 2018). Higher N availability can also affect the plants’ cell and physiological characteristics associated with drought and frost susceptibility (Carroll et al., 1999; Schaberg et al., 2002). Nitrogen input from agricultural practices, fossil fuel burning, and biomass burning can reach high latitudes (Forsius et al., 2010; Karlsson et al., 2013) and make sub-arctic plants more vulnerable to the temperature variability of WW events (Power et al., 1998; Phoenix et al., 2012). However, it is unclear which plant types will be most vulnerable to the combination of high N availability and WW events and whether this will result in a vegetation regime shift.

To address the impact of the combined stressors of N input and WW events on the community composition of sub-arctic vegetation we initiated an experiment that combined these factors in sub-arctic Norway. The simulation of WW events was done during three consecutive winters in February (2014–2016), while N additions were applied during the summer months. However, during the growing season directly following the first WW simulation (2014), the field sites were subject to intense grazing by caterpillars of the geometrid moths *Epirrita autumnata* and *Operophtera brumata* (Bjerke et al., 2017b, 2018; Pepi et al., 2017). Such grazing pressure greatly affect the growth response of dwarf shrubs, as these are targeted by the caterpillars that drop onto the ground cover once the birch trees have been defoliated (Lehtonen and Heikkinen, 1995; Malmström and Raffa, 2000; Jepsen et al., 2013; Karlsen et al., 2013). Grazing pressure can result in sub-arctic vegetation regime shifts where dominant dwarf shrubs are suppressed and herbs and grasses emerge (Tømmervik et al., 2004; Olofsson et al., 2013). This therefore resulted in a factorial field experiment where the impacts on sub-arctic heath vegetation of +N and WW simulations were compared but also included intense grazing by caterpillars across all treatments. We hypothesized that (1) summer N additions will negatively impact the winter plant physiological adaptations to frost and drought. Because N additions are known to exacerbate other stressors (Power et al., 1998; Phoenix et al., 2012) we expect N additions to result in an increase of the damage caused by WW events and grazing impact. The grazing and WW effects will be strongest on evergreen dwarf shrubs because this plant type appears most vulnerable to WW events (Bokhorst et al., 2015, 2018) while also being heavily grazed when caterpillars fall to the ground (Jepsen et al., 2013; Karlsen et al., 2013). Therefore, we hypothesize that in response to these extreme events (2) the plant community may start to shift from an evergreen dwarf shrub dominated community to one with higher dominance of cryptogams, grasses, herbs and deciduous plants with potential greater turnover of carbon flux rates and limiting carbon sequestration of these sub-arctic ecosystems.

## Materials and Methods

The study site was located on the small island Håkøya, situated in the fiord Balsfjorden between the larger island Kvaløya and the mainland (Tromsø, Norway, 69.66° N 18.78° E, 30 m a.s.l.). The western part of Håkøya, where this study was located, has a mosaic of open deciduous woodland dominated by *Betula pubescens* Ehrh. and treeless heathland dominated by the dwarf shrub *E. nigrum* L. The climate is relatively mild for these latitudes due to the warm Norwegian current (which is a branch of the North Atlantic current), resulting in mean summer and winter temperatures of 12°C and -4°C, respectively (Førland et al., 2009). Annual precipitation is ca. 1000 mm and the winter snowpack typically reach 60–80 cm depth. The experimental site was situated in an area with sparse distribution of birch trees (*B. pubescens*) and dominated by *E. nigrum* with a dense cover of the moss *Pleurozium schreberi* (Willd. ex Brid.) Mitt. Sub-dominant plant species included *Vaccinium vitis-idaea* L.*, V. uliginosum* L.*, V. myrtillus* L*, Cornus suecica* L.*, Avenella flexuosa* (L.) Drejer, the moss *Polytrichum commune* Hedw. and *Cladonia* lichens.

The experiment consisted of 24 plots (1 m × 2 m) with four treatments replicated six times: control (C), N addition (+N), extreme winter warming events (WW), and WW with N addition (WW+N). Ammonium nitrate solutions (5 kg N ha^-1^) were applied by watering can (2 L volume) across each +N treatment plot three times during the growing season at monthly intervals. The N additions were at the lower limit of effect doses for most plants (Phoenix et al., 2012) and chosen to reflect realistic scenarios of N input resulting from airborne transport for these sub-arctic regions (Karlsson et al., 2013). The WW simulations were implemented by using four infrared heaters (800 W emitting at 3 μm; HS 2408, Kalglo Electronics Co., Bethlehem, PA, United States) that were suspended in parallel (65 cm apart) from wooden frames. This produced a thermal radiation flux of 270 W m^-2^ to the plots (at zero wind speed). Lamps were on between 9 and 16 February 2014, 13–20 February 2015, and 11–18 February 2016. The snow pack (60–80 cm deep) gradually melted out during 3 days after which the lamps remained on for another 4 days. Lamps were adjusted in height above the surface to achieve leaf temperatures of ca. 5°C. Leaf temperatures were monitored at least twice per day by obtaining a reading of a thermocouple attached to the underside of a plant leaf in each plot. Lamps were turned off after 7 days and removed from the frames. The experimental plots were left exposed to ambient conditions and build-up of a new snowpack for the remainder of the winter. We marked out an additional 12 quadrats (1 m × 1 m) which were treated as control (*n* = 6) and nitrogen additions (*n* = 6) from which we could sample winter plant tissues and measure ecosystem gas fluxes without disturbing the main C and +N experimental plots. Temperature at canopy height was monitored throughout the year by temperature loggers (Hobo UA-001-08, Onset Computer Corporation, MA, United States) recording at hourly intervals in four control plots and four plots exposed to extreme WW events. Loggers were shielded from direct sunlight by a white dome cover.

### Leaf Phenology and Vegetation Composition

Vegetative bud development was monitored during the subsequent growing season (early June onward). For this, 10 randomly selected shoots of the dwarf shrubs *E. nigrum, V. vitis-idaea*, and *V. myrtillus* were tagged in each plot and surveyed every week or more frequently depending on the speed of development. Due to the presence of large caterpillar numbers of geometrid moths, a large proportion (see results) of tagged shoots were grazed and new unaffected shoots were selected in spring 2014. Vegetative bud development was recorded by noting when the bud had burst and the first leaf had fully expanded (Bokhorst et al., 2008). During the 2015 WW event we quantified bud elongation of *V. myrtillus*, as it represents a first step in bud development (Bokhorst et al., 2010). The abundance and cover of plant species was quantified using the point intercept method in a quadrant (30 cm × 30 cm) in the middle of each plot during peak plant biomass (August). A total of 121 point counts at 2.5 cm intervals were made of the vegetation in each square by counting the number of times a vertical pin touched plant parts (Jonasson, 1988). Cryptogams were counted as present or absent, while vascular plants could be hit more than once by each vertical pin. For *E. nigrum* only shoots were counted rather than every leaf hit to avoid overrepresentation due to the high number of tightly packed, small needle-like leaves. Point intercept counts of vascular plants were converted to biomass using regression formulas (Supplementary Table S1) (Jonasson, 1988; Bokhorst et al., 2011). Species cover was quantified from point count survey based on presence or absence at each point. Changes in plant biomass and cover were compared from 1 year to the next starting at August 2013.

### Normalized Difference Vegetation Index (NDVI)

As a measure of vegetation activity (“health”) across the experimental plots we quantified NDVI by using two different handheld proximate sensor. We used a Maxmax-modified Canon camera (LDP LLC, Carlstadt, NJ, United States) where an infrared sensor replaced the normal sensor (the blue channel records the visible light and the red channel the near infrared). In addition, we used a GreenSeeker (Trimble Navigation Ltd., Sunnyvale, CA, United States) which generates its own radiation for NDVI measurements, while the MaxMax camera is a passive instrument, using reflected and incident radiation (Sakamoto et al., 2012). Plot pictures were taken during peak vegetation biomass, the second week of August, each year (2013–2016). During 2015 and 2016, the two types of NDVI measurements were also done at weekly intervals to monitor changes in plot-level greenness during the growing season (May–August).

### CO_2_ Fluxes

Respiration rates of *V. vitis-idaea* (individual leaves) and shoots (2 cm) of *E. nigrum* (2014 and 2015) and the moss *P. schreberi* (2015 only) were quantified on an Infrared Gas Analyser (IRGA) (GFS-3000, Walz GmbH, Effeltrich, Germany). Measurements were done in closed cuvettes in complete darkness, at 7000 ppm H_2_O, with 380-ppm base level of CO_2_ and temperature of the measuring head kept at 5°C. We used the mean respiration rates of nine measurements taken at 15 s intervals for each sample. Samples were collected during the last day of warming, after the maximum exposure period, and 3 days after the lamps had been turned off.

Ecosystem CO_2_ fluxes were measured during the WW events and in the following growing seasons (2014–2016) from May till August. Measurements were made by placing a clear chamber (20 cm × 20 cm × 20 cm) made from poly-methyl methacrylate over the vegetation and monitoring the rate of change in headspace CO_2_ concentration, across nine measurements at 10 s intervals, using an IRGA (EGM-4 PP Systems, Amesbury, MA, United States). To minimize internal chamber air exchange with the external environment, plastic skirts (20 cm wide) were attached to a square frame – onto which the chamber could be attached – and weighed down with chains (and snow in winter). The square frame was slotted onto four metal pins that were fixed in the plots to ensure that CO_2_ fluxes were measured at the same spot in each plot. Snow was removed 2 h before CO_2_ measurements from the additional C and +N plots, to allow any build-up of CO_2_ underneath the snow layer to diffuse out (Grogan and Jonasson, 2006).

### Plant Physiological Measurements

As a measure of winter plant physiological activity we quantified potential activity of PSII using a mini-PAM (Walz, Effeltrich, Germany) for *E. nigrum* and *V. vitis-idaea* using leaf clips, in the experimental warmed plots and from underneath the snow as controls during early morning (06:00–06:30) when the sun was below the horizon and PAR levels were zero. Measurements were done during the last 2 days of warming when plants had the longest exposure to the warming, to quantify potential release from winter dormancy, and in addition 4 days after the lamps had been turned off to quantify the response of PSII following freezing. One leaf (*V. vitis-idaea*) or shoot (*E. nigrum*) was measured in each experimental plot during each measuring day.

To quantify changes in the fatty acid composition of the cell membranes as a result of the warming and freezing, we collected leaf samples (*n* = 5), while plants had been exposed to WW for 4 days and 4 days after the WW treatment had been turned off. Samples from control plots were retrieved during either of these sampling events. All samples were brought back to the laboratory (within 1–2 h) and frozen at –20°C, freeze-dried, ground, and analyzed for fatty acids following the direct methylation procedure (Browse et al., 1986). Samples of 5–20 mg were dissolved in 1 mL methanolic hydrochloric acid (HCl) (1M) and an internal standard (heptadecanoic acid, C17:0) was added to a glass tube. The solution was heated to 80–100°C for 1 h, and after cooling, 0.4 mL hexane and 1 mL of 0.9% sodium chloride (NaCl) were added to each sample. The fatty acid methyl esters (FAMES) were extracted into the hexane phase by vigorous shaking. The tubes were centrifuged for 10 min to separate the phases completely, and a sample was then taken directly from the hexane phase. Samples were stored at –20°C until gas chromatography (GC) analyses, according to Mæhre et al. (2013). The instrument used was an Agilent 6890N equipped with a flame ionization detector (FID) (Agilent Technologies Inc., Santa Clara, CA, United States) and a CP7419 capillary column (50 m × 250 μm × 0.25 μm nominal, Varian Inc., Middelburg, Netherlands). The fatty acids were identified by comparing against the commercial fatty acid standards PUFA 1, 2, and 3 (Sigma-Aldrich Chemicals Co., St. Louis, MO, United States) and the GLC standards 80, 411, and 412 (NuChec Prep. Inc., Elysian, MN, United States). The amount of each fatty acid was calculated by comparing peak area with the known amount of an internal standard (C17:0). The proportions of the single fatty acids were used in further analysis. We calculated the unsaturation to saturation ratio (U/S ratio) as the ratio between the total proportion of all unsaturated fatty acids and the total proportion for all saturated fatty acids (van Dooremalen et al., 2011). The detected fatty acid composition differed greatly between plant species, which limited direct comparisons of specific fatty acids between study species. However, changes in fatty acid composition could be quantified in response to the treatments.

### Calculations and Statistical Analyses

We used repeated measures ANOVA to detect differences in leaf phenology, NDVI, and CO_2_ fluxes between treatments (WW and +N) during the growing seasons of 2014–2016. To identify changes in plant biomass and cryptogam cover across the measurement years (2013–2016) we used a linear mixed effects model with treatments (WW *vs.* C and –N *vs.* +N) and years as fixed factors and block as a random factor. *P*-values were obtained by likelihood ratio tests of the full model with the effect in question against the model without the effect in question. A Principal Component Analyses (PCA) was used to summarize changes in vegetation composition of vascular plant and cryptogam cover between treatments, and the first two PCs were analyzed in the same way as the plant biomass. Treatment effects on fatty acid concentrations and U/S ratio were tested using a factorial ANOVA with Treatment and +N as fixed factors. We used Tukey HSD tests at *P* = 0.05 to identify differences in means between WW and C (with and without +N) whenever the interaction effect was significant. A visual inspection did not show any patterns in the residuals. All statistical analyses were carried out using R 3.3.0 (R Core Team, 2015).

## Results

### Temperature Effects of Winter Warming Treatment

**FIGURE 1:**
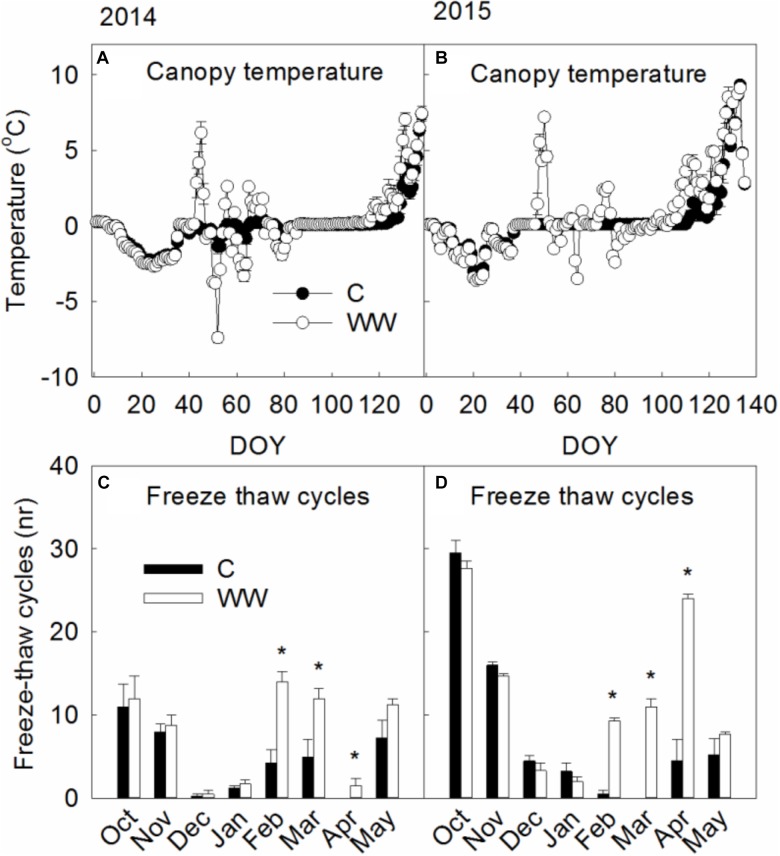
**(A,B)** Show canopy temperatures measured during 2014 and 2015 respectively in control plots **(C)** and those exposed to extreme winter warming (WW). **(C,D)** Show the monthly number of freeze-thaw cycles during 2014 and 2015 respectively. Mean daily canopy temperatures and monthly number of freeze thaw cycles during the winters of 2014 and 2015. Data points and bars are means of *n* = 4 with SE as error bars. ^∗^ indicate significant (*P* < 0.05) differences between treatments. Canopy temperatures were measured following the extreme winter warming event of 2016 but not during the winter period before therefore, these are not shown.

Canopy temperature increased to 6.3–7.2°C in the WW plots while canopy temperatures underneath the snow were around freezing (-0.1–0.1°C) in the control plots (Figure 1). Minimum temperatures were somewhat lower in WW compared to C during 2014 (-11.8°C vs. -9.7°C) and 2016 (-6.6°C vs. -1.0°C) but did not differ during 2015 (-9.1°C). The number of freeze thaw cycles increased following WW by 67% and 57% during 2014 and 2015, respectively, and from 1 in C to 29 in WW during 2016 (Figures 1C,D). Leaf and moss temperatures were on average 5 and 10°C, respectively, during the WW treatments.

### Winter Physiology and Ecosystem Responses

Efficiency of Photosystem II (Fv/Fm) of *E. nigrum* increased (*P* < 0.001) by 77 and 80% in the WW (0.59) and WW+N (0.59) treatments, respectively, as compared to C (0.33) during 2014, indicating a release from winter dormancy. Fv/Fm of *V. vitis-idaea* increased (*P* < 0.01) by 26 and 33% in the WW (0.61) and WW+N (0.64) treatment, respectively, compared to C (0.48) during 2014. Fv/Fm no longer differed between the treatments after the warming lamps was turned off. Fv/Fm was unaffected by WW during 2015, and values across treatments were 0.68, 0.65, and 0.69 for *E. nigrum, V. vitis-idaea*, and *P. schreberi*, respectively. Winter leaf respiration decreased by 58% for *E. nigrum* (*F*_3,19_ = 3.8, *P* = 0.027) and with 43% for *P. schreberi* (*F*_3,19_ = 6.4, *P* = 0.005) in WW compared to C during 2015 but did not differ after lamps were turned off, and there were no differences between WW and WW+N. *Vaccinium vitis-idaea* leaf respiration was unaffected by the treatments (*F*_3,19_ = 2.0, *P* = 0.155).

Bud length of *V. myrtillus* increased by 12% during the 2015 warming event, representing an increase of 0.14 mm and 0.17 mm in WW and WW+N, respectively.

Winter ecosystem respiration was twice as high in WW (WW: 14.0 ± 1.9 mg C m^-2^h^-1^ and WW+N:16.9 ± 4.1 mg C m^-2^h^-1^) compared to C (6.4 ± 2.6 mg C m^-2^ h^-1^) during the last day of warming in 2014 (*F*_2,9_ = 9.8, *P* = 0.005), but no respiration differences were found during the 2015 WW event (*F*_3,2_ = 7.0, *P* = 0.074). There was no effect of +N on winter ecosystem CO_2_ fluxes. Snow fungi were observed in the plots during 2014 (but not quantified), while during 2015, snow fungi covered 26% (SE: 4.3) and 47% (9.1) of the surface of WW and WW+N (*F*_1,9_ = 3.7, *P* = 0.088).

### Membrane Fatty Acids

**FIGURE 2:**
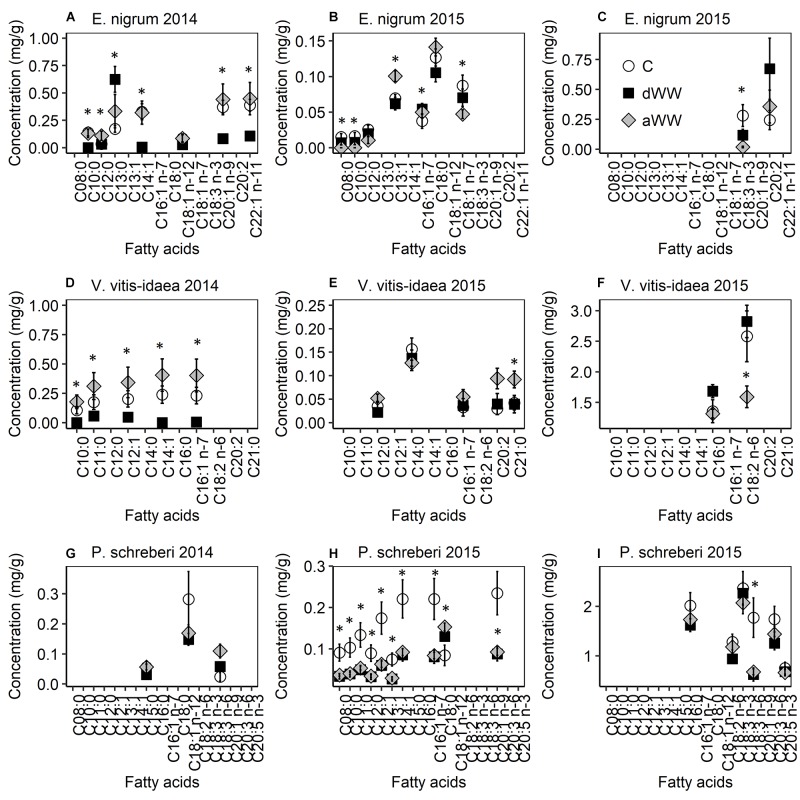
Composition of cell membrane fatty acids of *Empetrum nigrum* **(A–C)**, *Vaccinium vitis-idaea* **(D–F)**, and *Pleurozium schreberi* **(G–I)** measured during (dWW) and after (aWW) extreme winter warming events in 2014 and 2015. Note that for clarity the 2015 fatty acids are separated by concentration. Data points are means of five replicate samples with SE as error bars. Asterix indicate significant (*P* < 0.05) difference in fatty acid concentrations between extreme winter warming and control plots.

The ratio of unsaturated to saturated fatty acids was not affected by the treatments for any of the study species during 2014 or 2015 (data not shown). The concentration of various membrane fatty acids declined for *E. nigrum, V. vitis-idaea*, and *P. schreberi* in response to WW during 2014 (Supplementary Table S2 and Figure 2). However, during the second WW event (2015), the response varied between treatments and species and not all fatty acids were found during both years. Five affected fatty acids were consistently lower (from 3 to 100%) during the WW of 2014 in *E. nigrum* compared to control plots and following the WW (Figure 2A). However, C13:0 was increased (263%) during WW compared to control plots. The fatty acids C16:1n-7, C18:1n-7, and C18:3n-3 were all lower following the 2015 WW compared to control plots, while C8:0, C10:0, and C12:0 were not found in detectable concentrations. C13:1 had highest concentrations (45% higher) following WW compared to control samples (Figures 2B,C). Nitrogen increased C18:1 n-12 by more than three times during 2014 while C12:0 was reduced by 69% during 2015. C18:0 and C20:2 (eicosadienoic acid) responded to WW in combination with +N; for C18:0 there were no Tukey *post hoc* differences, while C20:2 concentrations were consistently higher during WW without N compared to the other treatments.

Affected fatty acids (*n* = 5) were consistently lower (from 67 to 100%) during the WW of 2014 in *V. vitis-idaea* compared to frozen samples from control plots and following the WW (Figure 2D). During 2015, C18:2 n-6 declined by 38% following the WW event compared to control samples, while C21:0 and C20:2 increased by 219 and 118%, respectively, following the WW event (Figures 2E,F). The remaining near-significant differences (*P* > 0.05) in fatty acid concentrations were found during and after the WW event. Nitrogen did not affect fatty acid concentrations during 2014. During 2015, C21:0 and C20:2 were both reduced (58 and 54%, respectively) when +N was applied. C14:0 had a significant WW × N interaction, but Tukey *post hoc* testing did not indicate significant treatment differences (Supplementary Table S2).

There were three affected fatty acids (C15:0, C18:1 n-12, and C18:3 n-6) responding to the interaction between WW and +N during 2014 in *P. schreberi* but all in a different way (Figure 2G). Concentrations of C15:0 were approximately 10 times higher in C +N compared to C without N but not compared to any of the other treatments. Concentrations of C18:1 n-12 were highest in C (without +N) compared to all other treatments, while concentrations of C18:3 n-6 were highest for WW+N compared to all other treatments. During the winter of 2015, the majority of the affected fatty acids (*n* = 16) were consistently higher (9–65%) in control plots, while only C18:1 n-12 was higher during and after the WW event (54 and 81%, respectively) (Figures 2H,I). Nitrogen increased the concentration of C20:3 n-6 and C20:5 n-3 by 31% during 2015, while the fatty acids C16:0, C18:2 n-6, C18:3 n-3, and C20:3 n-6 had the highest concentrations in the control plots that also received +N.

### Ecosystem Responses in the Growing Season

Leaf emergence timing was not affected by the treatments during any of the years, although there were differences in the percentage of emerged leaves between treatments across the measuring periods (Supplementary Figure S1 and Table 1). *Empetrum nigrum* had higher total percentage of emerged leaves in WW (65%) compared to control (33%) at the final measuring date in 2014. There were no treatment effects on *V. myrtillus* leaf emergence during any of the years. While *V. vitis-idaea* had highest percentage (40%) of total fully developed leaves in the +N treatment at the final measuring dates in 2016.

**Table 1:** Repeated measures ANOVA results (*F*-values) of leaf phenology, plot NDVI values and CO_2_ gas fluxes in response to extreme winter warming events (WW), nitrogen additions (N) and their combination during three growing seasons.^∗^<0.05, ^∗∗^<0.01, ^∗∗∗^<0.001.

		N	WW	Date	N:WW	N:Date	WW:Date	N:WW:Date
*E. nigrum*	2014	0.0	4.2	77.6***	0.5	0.0	3.6*	0.7
	2015	0.4	0.2	14.7***	0.7	0.6	0.4	0.4
	2016	0.1	0.9	50.8***	0.1	1.8	2.3	0.1
*V. myrtillus*	2014	0.5	0.3	30.7***	0.1	0.5	1.2	0.4
	2015	1.3	1.2	18.4***	0.4	1.1	1.4	0.4
	2016	1.3	0.2	44.1***	0.5	2.1	0.4	0.4
*V. vitis-idaea*	2014	5.7*	0.1	1.1	1.0	0.5	1.1	0.1
	2015	0.0	0.0	150.3***	0.3	2.4	0.5	1.2
	2016	1.6	1.0	53.0***	3.1	2.8*	1.5	0.6
NDVI	2013	0.8	3.4		0.1			
	2014	0.8	0.2		0.7			
	2015	1.2	0.9	4.5**	0.0	1.4	0.9	1.1
	2016	0.3	0.0	935.1***	0.8	0.7	3.0***	0.5
Greenseeker	2015	1.9	0.3	58.8***	0.0	0.4	0.0	0.5
	2016	3.7	0.6	255.6***	1.8	0.5	1.2	0.8
ER	2014	1.3	3.1	96.7***	0.0	1.6	0.9	0.5
	2015	0.0	0.4	10.3***	0.0	0.2	0.6	0.3
	2016	0.0	0.4	10.3***	0.0	0.2	0.6	0.3
NPP	2014	1.9	2.0	12.8***	0.9	2.7**	1.1	1.4
	2015	1.0	1.2	0.9	0.3	0.7	0.2	1.5
	2016	0.7	3.6	31.0***	0.3	0.5	1.2	0.2
GPP	2014	0.0	3.9	48.1***	0.3	2.6**	1.0	1.0
	2015	0.4	1.1	7.2***	0.1	0.1	0.4	0.1
	2016	0.0	3.2	23.6***	0.2	0.3	0.4	0.3

**FIGURE 3:**
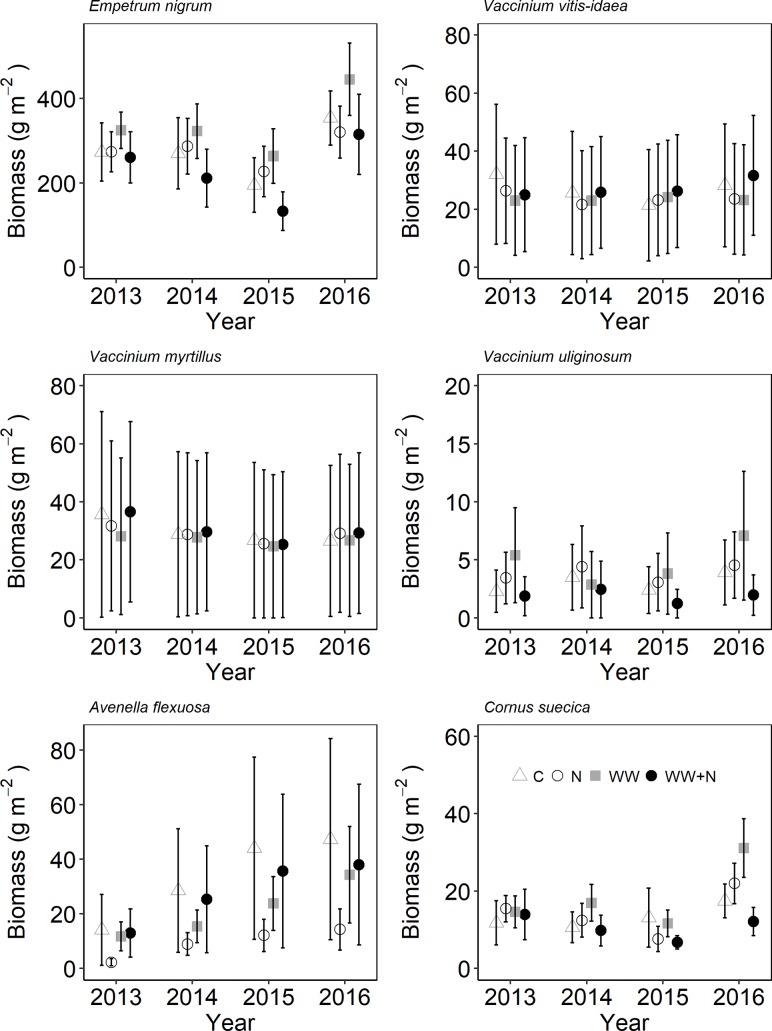
Species-specific plant biomass following extreme winter warming (WW) and nitrogen (N) additions in sub-arctic Norway. Data points are mean of *n* = 6 plots with SE as error bars.

**FIGURE 4:**
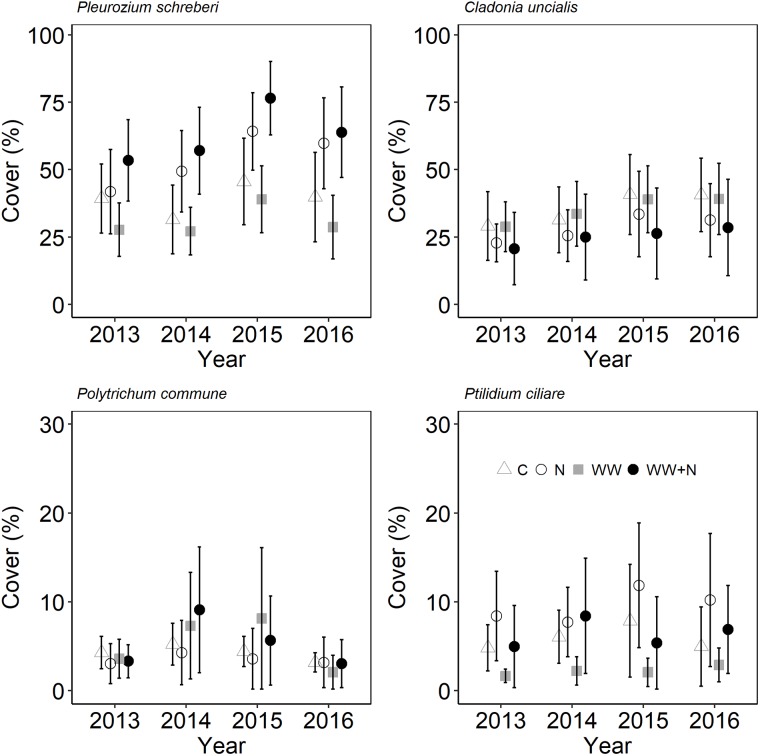
Species-specific cryptogam cover following extreme WW and nitrogen (N) additions in sub-arctic Norway. Data points are mean of *n* = 6 plots with SE as error bars.

Plant biomass and cryptogam cover were not affected by WW, +N or their interaction (Table 2). Instead, biomass of most vascular plant species declined following the second WW events in all plots (including C) due to the caterpillar outbreak. Especially the dwarf shrubs were targeted by the caterpillars resulting in a large proportion of grazed shoots of *E. nigrum* (81%), *V. myrtillus* (87%), and *V. vitis-idaea* (17%) during 2014. There were no significant differences in the number of grazed shoots between treatments. *Avenella flexuosa* and *C. suecica* biomass were unaffected by the treatments and did not appear to be affected by caterpillar grazing. Plant biomass increased again during the last (2016) growing season (Figure 3). Moss cover (*P. schreberi* and *P. commune*) reached its peak following the second WW event (summer 2015) but declined again in 2016 (Figure 4). The lichen *Cladonia uncialis* and the liverwort *Ptilidium ciliare* had a stable cover between years and were unaffected by the treatments (Table 2 and Figure 4). The PCA analysis indicated that the vegetation community did not change between treatments or years (Table 2).

**Table 2:** Mixed-effect model output of changes in plant biomass (number of point intercept hits) and cryptogam cover between years in response to extreme winter warming events (WW) and nitrogen additions.

	WW		Nitrogen		WW × N		Year	
	χ^2^	*P*	χ^2^	*P*	χ^2^	*P*	χ^2^	*P*
**Vascular plants (Biomass change %)**					
*Cornus suecica*	3.8	0.148	2.9	0.233	2.8	0.093	30.7	<0.001
*Avenella flexuosa*	4.1	0.130	3.3	0.194	1.2	0.268	3.9	0.144
*Empetrum nigrum*	2.5	0.285	2.4	0.305	2.4	0.124	50.1	<0.001
*Vaccinium myrtillus*	5.1	0.080	0.4	0.803	0.2	0.632	24.7	<0.001
*Vaccinium uliginosum*	0.1	0.933	0.8	0.656	0.1	0.886	7.5	0.024
*Vaccinium vitis-idaea*	1.0	0.611	2.3	0.320	1.0	0.322	3.4	0.182
**Cryptogams (cover change)**					
*Cladonia uncialis*	0.1	0.999	0.2	0.927	0.0	0.969	4.3	0.119
*Pleurozium schreberi*	0.2	0.855	2.2	0.339	0.2	0.676	30.1	<0.001
*Polytrichum commune*	0.0	0.996	0.1	0.968	0.0	1.000	7.7	0.021
*Ptilidium ciliare*	0.1	0.979	0.1	0.945	0.1	0.898	1.3	0.526
PC1	2.9	0.238	3.8	0.152	2.7	0.103	0.2	0.641
PC2	1.0	0.603	1.0	0.596	1.0	0.318	0.2	0.659

Plot NDVI was not consistently affected by WW, +N or their interaction. During 2016 WW plots were 5% lower NDVI values compared to C in spring while later on in the season there was no difference (Table 1 and Supplementary Figure S2). NDVI plot values were on average 38% lower during August 2015 compared to starting values (Table 1). CO_2_ fluxes increased with time during the growing seasons (Table 1), but there were no differences in ecosystem ER, NPP, and GPP between the treatments during the growing seasons (Supplementary Figure S3), with the exception of some lower NPP values under +N treatment in 2016.

## Discussion

We anticipated a vegetation regime shift from evergreen dwarf shrubs (heath) to one of cryptogams, grasses, and deciduous plants (meadow) as a result of WW with this being exacerbated by the N treatment. Although evergreen dwarf shrubs were reduced in biomass following the grazing and WW events, the communities did not significantly differ in species composition between treatments and years, indicating a strong resistance to change in these *E. nigrum* dominated ecosystems. Furthermore, there was no strong evidence for any interaction between the multiple extreme events, indicating that multiple stresses do not necessarily lead to accumulation of biological stress responses (Thompson et al., 2008; Jackson et al., 2016).

We expected that +N would exacerbate the effects of WW and grazing pressure but no such responses were observed. We expected that N additions would promote plant growth, but simultaneously increase vulnerability to freezing stress due to changes in cell and physiological characteristics associated with drought and frost susceptibility (MacGillivray et al., 1995; Carroll et al., 1999; Schaberg et al., 2002). However, the added N-levels (5 kg ha^-1^) did not lead to measurable responses in the control plots suggesting that these coastal *E. nigrum* vegetation types should be able to withstand N depositions events (Forsius et al., 2010; Karlsson et al., 2013). In addition, mid-winter bud swelling, Fv/Fm and leaf respiration, indicators of a potential release from winter dormancy responded similarly to WW with or without +N. The moss *P. schreberi* did show changes in membrane fatty acids to +N, but not consistently in combination with WW. The fatty acid C20:2 increased in WW–N compared to WW+N for both *E. nigrum* and *V. vitis-idaea*, indicating that soil N availability may affect cell membrane adaptations to freezing stress. However, as we did not find consistent phenology and plant biomass responses to WW+N in the following growing seasons, it appears that the role of N on the plants’ winter physiology appears limited in this study. Nitrogen additions have been shown to increase insect damage to *Calluna vulgaris* communities (Power et al., 1998) but this was not observed in our study. Overall, the combination of stress events and low dose N deposition does not appear to seriously affect these coastal sub-arctic *E. nigrum* communities.

In support of our second hypothesis, the evergreen dwarf shrub *E. nigrum* declined in response to the grazing by geometrid caterpillars while the herb *C. suecica*, not often grazed upon, increased over the study period. *Empetrum nigrum* is frequently targeted by caterpillars when their main food plant birch trees have been defoliated (Jepsen et al., 2013), which explains the decline of this dwarf shrub over time. The increase of *C. suecica* was also found elsewhere in northern Fennoscandia as a combined effect of grazing pressure, increased precipitation and deposition of nitrogen through precipitation and caterpillar fecal matter (Tømmervik et al., 2004; Karlsen et al., 2013). However, we did not find any such response from N additions in our study, which indicates that the applied levels were insufficient to elicit a measurable plant growth response or that caterpillar fecal N input was much higher. In addition, moss cover was highest during 2015, reflecting the opening of the vascular plant cover canopy, as also observed following similar extreme events in physiognomically comparable open birch woodland in a more continental upland region of northern Scandinavia (Bokhorst et al., 2015). However, the evergreen species, such as *E. nigrum* and *V. vitis-idaea*, quickly recovered their biomass to starting levels in this study, indicating that this coastal *E. nigrum* dominated vegetation were fairly resistant to the combination of WW events, N additions and invertebrate grazing. The ecosystem CO_2_ fluxes measured during the growing season also did not show differences between treatment plots indicating that the plant community shifts were too limited to impact this ecosystems carbon balance (Luyssaert et al., 2007; De Deyn et al., 2008), although the 2016 values were notably higher than during the 2 previous years (Supplementary Figure S3).

The muted response from the plant community, and NDVI values to the extreme WW events, may result from the intensive grazing impact overall, including the control plots, which could have diluted the effect of the imposed winter stress. The relative importance of such events, and their interaction, in shaping sub-arctic plant communities remains a complex interplay depending in large part on intensity, timing and duration (Olofsson et al., 2013). Furthermore, sub-dominant species cover was not affected enough to affect plot communities dominated by *E. nigrum* and *P. schreberi*, while high shoot defoliation of dwarf shrubs recorded during 2014 could have been partly compensated later on in the season (Bokhorst et al., 2011). In addition, studies on *C. vulgaris*-dominated ecosystems show that plant community responses to multiple environmental drivers can be affected by successional stage and disturbance regimes across Europe (Kröel-Dulay et al., 2015). As such, the high dominance of *E. nigrum* in our field site may reflect a successional stage that is more resistant than stages where other species comprise a larger proportion of the community or these coastal habitats have plants with greater physiological adaptability to cope with multiple stressors than habitats with more stable climates. With respect to the WW events, more pronounced plant responses have been reported when temperatures were lower (Bokhorst et al., 2009; Bjerke et al., 2017b), which is an important factor for frost drought-induced plant mortality (Kreyling et al., 2008; Bokhorst et al., 2010, 2018). The relative mild freezing temperatures may have allowed for winter physiological adaptations by *E. nigrum* and *V. vitis-idaea*, such as the greater proportion of longer chain fatty acids (Dalmannsdóttir et al., 2001; Schaberg et al., 2002). The N addition levels were chosen at the lowest concentration effect-response by most plant communities (Phoenix et al., 2012) as a realistic scenario, while higher N levels for a longer duration (>3 years) may result in additive biological responses (Power et al., 1998; Phoenix et al., 2012). Similarly, grazing intensity on plants is not consistent during each herbivore outbreak (Olofsson et al., 2013) indicating that sub-arctic plant community responses to multiple extreme events may depend predominantly on the event with the strongest intensity.

## Conclusion

The findings indicate that coastal sub-arctic dwarf shrub plant communities and growing season CO_2_ fluxes appear largely unaffected to a combination of extreme WW events, grazing and nitrogen additions in summer. This response may in part be the result of inherent physiological adaptations, such as changing the membrane fatty acids (Dalmannsdóttir et al., 2001), during winter and the mild winter freezing temperatures (Bokhorst et al., 2018). The grazing impact appears to have overshadowed all plant responses to the WW and +N treatments indicating that multiple extreme events, that in theory can enhance each other’s effects (Power et al., 1998; Phoenix et al., 2012), do not necessarily increase biological stress responses.

## Author Contributions

SB and JB designed and conducted the experimental work. All authors contributed to the experimental design, field and laboratory work, wrote or commented on the main text and supplementary notes.

## Conflict of Interest Statement

The authors declare that the research was conducted in the absence of any commercial or financial relationships that could be construed as a potential conflict of interest.

## References

[B1] Bjerke J. W. Bokhorst S. Callaghan T. V. Phoenix G. K. (2017a). Persistent reduction of segment growth and photosynthesis in a widespread and important sub-arctic moss species after cessation of three years of experimental winter warming. *Funct. Ecol.* 31 127–134. 10.1111/1365-2435.12703

[B2] Bjerke J. W. Treharne R. Vikhamar-Schuler D. Karlsen S. R. Ravolainen V. Bokhorst S. (2017b). Understanding the drivers of extensive plant damage in boreal and arctic ecosystems: insights from field surveys in the aftermath of damage. *Sci. Total Environ.* 599–600, 1965–1976. 10.1016/j.scitotenv.2017.05.050 28558420

[B3] Bjerke J. W. Bokhorst S. Zielke M. Callaghan T. V. Bowles F. W. Phoenix G. K. (2011). Contrasting sensitivity to extreme winter warming events of dominant sub-arctic heathland bryophyte and lichen species. *J. Ecol.* 99 1481–1488. 10.1111/j.1365-2745.2011.01859.x

[B4] Bjerke J. W. Wierzbinski G. Tømmervik H. Phoenix G. K. Bokhorst S. (2018). Stress-induced secondary leaves of a boreal deciduous shrub (*Vaccinium myrtillus*) overwinter then regain activity the following growing season. *Nord. J. Bot.* 36:e01894 10.1111/njb.01894

[B5] Bokhorst S. Bjerke J. W. Bowles F. P. Melillo J. M. Callaghan T. V. Phoenix G. K. (2008). Impacts of extreme winter warming in the sub-Arctic: growing season responses of dwarf-shrub heathland. *Glob. Change Biol.* 14 2603–2612. 10.1111/j.1365-2486.2008.01689.x

[B6] Bokhorst S. Bjerke J. W. Davey M. Taulavuori K. Taulavuori E. Laine K. (2010). Impacts of extreme winter warming events on plant physiology in a sub-arctic heath community. *Physiol. Plant* 140 128–140. 10.1111/j.1399-3054.2010.01386.x 20497369

[B7] Bokhorst S. Bjerke J. W. Street L. Callaghan T. V. Phoenix G. K. (2011). Impacts of multiple extreme winter warming events on sub-arctic heathland: phenology, reproduction, growth, and CO2 flux responses. *Glob. Change Biol.* 17 2817–2830. 10.1111/j.1365-2486.2011.02424.x

[B8] Bokhorst S. Bjerke J. W. Tømmervik H. Callaghan T. V. Phoenix G. K. (2009). Winter warming events damage sub-arctic vegetation: consistent evidence from an experimental manipulation and a natural event. *J. Ecol.* 97 1408–1415. 10.1111/j.1365-2745.2009.01554.x

[B9] Bokhorst S. Jaakola L. Karppinen K. Edvinsen G. K. Mæhre H. K. Bjerke J. W. (2018). Contrasting survival and physiological responses of sub-arctic plant types to extreme winter warming and nitrogen. *Planta* 247 635–648. 10.1007/s00425-017-2813-6 29164366 PMC5809542

[B10] Bokhorst S. Phoenix G. K. Berg M. P. Callaghan T. V. Kirby-Lambert C. Bjerke J. W. (2015). Climatic and biotic extreme events moderate long-term responses of above- and belowground sub-arctic heathland communities to climate change. *Glob. Change Biol.* 21 4063–4075. 10.1111/gcb.13007 26111101

[B11] Browse J. McCourt P. J. Somerville C. R. (1986). Fatty acid composition of leaf lipids determined after combined digestion and fatty acid methyl ester formation from fresh tissue. *Anal. Biochem.* 152 141–145. 10.1016/0003-2697(86)90132-6 3954036

[B12] Carroll J. A. Caporn S. J. M. Cawley L. Read D. J. Lee J. A. (1999). The effect of increased deposition of atmospheric nitrogen on *Calluna vulgaris* in upland britain. *New Phytol.* 141 423–431. 10.1046/j.1469-8137.1999.00358.x

[B13] Crain C. M. Kroeker K. Halpern B. S. (2008). Interactive and cumulative effects of multiple human stressors in marine systems. *Ecol. Lett.* 11 1304–1315. 10.1111/j.1461-0248.2008.01253.x 19046359

[B14] Dalmannsdóttir S. Helgadottir A. Gudleifsson B. E. (2001). Fatty acid and sugar content in white clover in relation to frost tolerance and ice-encasement tolerance. *Ann. Bot.* 88 753–759. 10.1093/annbot/88.suppl_1.753

[B15] De Deyn G. B. Cornelissen J. H. C. Bardgett R. D. (2008). Plant functional traits and soil carbon sequestration in contrasting biomes. *Ecol. Lett.* 11 516–531. 10.1111/j.1461-0248.2008.01164.x 18279352

[B16] Førland E. Flatøy F. Hanssen-Bauer I. Haguen J. E. Isakesen K. Sorteberg A. (2009). Climate development in north norway and the svalbard region during 1900-2100. Tromsø. *Nor. Polar Inst. Ser.* 128:45.

[B17] Forsius M. Posch M. Aherne J. Reinds G. J. Christensen J. Hole L. (2010). Assessing the impacts of long-range sulfur and nitrogen deposition on arctic and sub-arctic ecosystems. *Ambio* 39 136–147. 10.1007/s13280-010-0022-7 20653276 PMC3357685

[B18] Grogan P. Jonasson S. (2006). Ecosystem CO2 production during winter in a swedish subarctic region: the relative importance of climate and vegetation type. *Glob. Change Biol.* 12 1479–1495. 10.1111/j.1365-2486.2006.01184.x

[B19] Jackson M. C. Loewen C. J. G. Vinebrooke R. D. Chimimba C. T. (2016). Net effects of multiple stressors in freshwater ecosystems: a meta-analysis. *Glob. Change Biol.* 22 180–189. 10.1111/gcb.13028 26149723

[B20] Jepsen J. U. Biuw M. Ims R. A. Kapari L. Schott T. Vindstad O. P. L. (2013). Ecosystem impacts of a range expanding forest defoliator at the forest-tundra ecotone. *Ecosystems* 16 561–575. 10.1007/s10021-012-9629-9

[B21] Jepsen J. U. Hagen S. B. Ims R. A. Yoccoz N. G. (2008). Climate change and outbreaks of the geometrids *Operophtera brumata* and *Epirrita autumnata* in subarctic birch forest: evidence of a recent outbreak range expansion. *J. Anim. Ecol.* 77 257–264. 10.1111/j.1365-2656.2007.01339.x 18070041

[B22] Jonasson S. (1988). Evaluation of the point intercept method for the estimation of plant biomass. *Oikos* 52 101–106. 10.2307/3565988

[B23] Karlsen S. Jepsen J. Odland A. Ims R. Elvebakk A. (2013). Outbreaks by canopy-feeding geometrid moth cause state-dependent shifts in understorey plant communities. *Oecologia* 173 859–870. 10.1007/s00442-013-2648-1 23568711 PMC3824357

[B24] Karlsson P. E. Ransijn J. Schmidt I. K. Beier C. De Angelis P. de Dato G. (2013). Biomass burning in eastern europe during spring 2006 caused high deposition of ammonium in northern fennoscandia. *Environ. Pollut.* 176 71–79. 10.1016/j.envpol.2012.12.006 23416271

[B25] Kivinen S. Rasmus S. Jylha K. Laapas M. (2017). Long-term climate trends and extreme events in northern fennoscandia (1914-2013). *Climate* 5:17 10.3390/cli5010016

[B26] Kreyling J. Wenigmann M. Beierkuhnlein C. Jentsch A. (2008). Effects of extreme weather events on plant productivity and tissue die-back are modified by community composition. *Ecosystems* 11 752–763. 10.1007/s10021-008-9157-9

[B27] Kröel-Dulay G. Ransijn J. Schmidt I. K. Beier C. De Angelis P de Dato G (2015). Increased sensitivity to climate change in disturbed ecosystems. *Nat. Commun.* 6:6682. 10.1038/ncomms7682 25801187

[B28] Lehtonen J. Heikkinen R. K. (1995). On the recovery of mountain birch after *Epirrita* damage in finnish lapland, with a particular emphasis on reindeer grazing. *Ecoscience* 2 349–356. 10.1080/11956860.1995.11682303

[B29] Liess M. Foit K. Knillmann S. Schäfer R. B. Liess H.-D. (2016). Predicting the synergy of multiple stress effects. *Sci. Rep.* 6:32965. 10.1038/srep32965 27609131 PMC5017025

[B30] Liston G. E. Hiemstra C. A. (2011). The changing cryosphere: pan-arctic snow trends (1979-2009). *J. Climate* 24 5691–5712. 10.1175/JCLI-D-11-00081.1

[B31] Luyssaert S. Inglima I. Jung M. Richardson A. D. Reichstein M. Papale D. (2007). CO2 balance of boreal, temperate, and tropical forests derived from a global database. *Glob. Change Biol.* 13 2509–2537. 10.1111/j.1365-2486.2007.01439.x

[B32] MacGillivray C. W. Grime J. P. The Integrated Screening Programme (ISP) Team. (1995). Testing predictions of the resistance and resilience of vegetation subjected to extreme events. *Funct. Ecol.* 9 640–649. 10.2307/2390156

[B33] Malmström C. M. Raffa K. F. (2000). Biotic disturbance agents in the boreal forest: considerations for vegetation change models. *Glob. Change Biol.* 6 35–48. 10.1046/j.1365-2486.2000.06012.x 35026937

[B34] Mæhre H. K. Hamre K. Elvevoll E. O. (2013). Nutrient evaluation of rotifers and zooplankton: feed for marine fish larvae. *Aquac. Nutr.* 19 301–311. 10.1111/j.1365-2095.2012.00960.x

[B35] Ögren E. (1996). Premature dehardening in *Vaccinium myrtillus* during a mild winter: a cause for winter dieback? *Funct. Ecol.* 10 724–732. 10.2307/2390507

[B36] Olofsson J. te Beest M. Ericson L. (2013). Complex biotic interactions drive long-term vegetation dynamics in a subarctic ecosystem. *Philos. Trans. R. Soc. B Biol. Sci.* 368:20120486. 10.1098/rstb.2012.0486 23836791 PMC3720058

[B37] Pepi A. A. Vindstad O. P. L. Ek M. Jepsen J. U. (2017). Elevationally biased avian predation as a contributor to the spatial distribution of geometrid moth outbreaks in sub-arctic mountain birch forest. *Ecol. Entomol.* 42 430–438. 10.1111/een.12400

[B38] Phoenix G. K. Emmett B. A. Britton A. J. Caporn S. J. M. Dise N. B. Helliwell R. (2012). Impacts of atmospheric nitrogen deposition: responses of multiple plant and soil parameters across contrasting ecosystems in long-term field experiments. *Glob. Change Biol.* 18 1197–1215. 10.1111/j.1365-2486.2011.02590.x

[B39] Power S. A. Ashmore M. R. Cousins D. A. Sheppard L. J. (1998). Effects of nitrogen addition on the stress sensitivity of *Calluna* vulgaris. *New Phytol.* 138 663–673. 10.1046/j.1469-8137.1998.00160.x

[B40] R Core Team. (2015). *R: A Language and Environment for Statistical Computing*. Vienna: R Foundation for Statistical Computing.

[B41] Sakamoto T. Gitelson A. A. Nguy-Robertson A. L. Arkebauer T. J. Wardlow B. D. Suyker A. E. (2012). An alternative method using digital cameras for continuous monitoring of crop status. *Agric For.Meteorol.* 154-155 113–126. 10.1016/j.agrformet.2011.10.014

[B42] Schaberg P. G. DeHayes D. H. Hawley G. J. Murakami P. F. Strimbeck G. R. McNulty S. G. (2002). Effects of chronic N fertilization on foliar membranes, cold tolerance, and carbon storage in montane red spruce. *Can. J. For. Res.* 32 1351–1359. 10.1139/x02-059

[B43] Schaberg P. G. Shane J. B. Hawley G. J. Strimbeck G. R. DeHayes D. H. Cali P. F. (1996). Physiological changes in red spruce seedlings during a simulated winter thaw. *Tree Physiol.* 16 567–574. 10.1093/treephys/16.6.567 14871710

[B44] Thompson P. L. St-Jacques M.-C. Vinebrooke R. D. (2008). Impacts of climate warming and nitrogen deposition on alpine plankton in lake and pond habitats: an in vitro experiment. *Arctic Antarctic Alpine Res.* 40 192–198. 10.1657/1523-0430(06-105)[THOMPSON]2.0.CO;2

[B45] Tømmervik H. Johansen B. Tombre I. Thannheiser D. Høgda K. A. Gaare E. (2004). Vegetation changes in the nordic mountain birch forest: the influence of grazing and climate change. *Arctic Antarctic Alpine Res.* 36 323–332. 10.1657/1523-0430(2004)036[0323:VCITNM]2.0.CO;2

[B46] van Dooremalen C. Suring W. Ellers J. (2011). Fatty acid composition and extreme temperature tolerance following exposure to fluctuating temperatures in a soil arthropod. *J. Insect. Physiol.* 57 1267–1273. 10.1016/j.jinsphys.2011.05.017 21704631

[B47] Vikhamar-Schuler D. Isaksen K. Haugen J. E. Tømmervik H. Luks B. Schuler T. V. (2016). Changes in winter warming events in the nordic arctic region. *J. Climate* 29 6223–6244. 10.1175/JCLI-D-15-0763.1

