# Supplementary material for: Impact of Multiple Ecological Stressors on a Sub-Arctic Ecosystem: No Interaction Between Extreme Winter Warming Events, Nitrogen Addition and Grazing
Source: Front Plant Sci. 2018 Nov 30;9:1787. doi: 10.3389/fpls.2018.01787 (PMC6284199; doi:10.3389/fpls.2018.01787)

**Supplementary information**

Table S1. Regression equations used for calculating plant biomass from point intercept hits. Data obtained from vegetation surveys conducted at Abisko in sub-Arctic Sweden. Note that that there was limited replicate data for herbs and grasses and these were therefore pooled.

| Species | regression equation | r^2^ | (n) |
| --- | --- | --- | --- |
| *Cornus suecica* | 0.0901x - 0.036 | 0.778 | 6 |
| *Avenella flexuosa* | 0.0901x - 0.036 | 0.778 | 6 |
| *Empetrum nigrum* | 0.1932x + 1.5238 | 0.988 | 20 |
| *Vaccinium myrtillus* | 0.077x + 2.2193 | 0.604 | 6 |
| *Vaccinium uliginosum* | 0.0284x + 0.0637 | 0.394 | 10 |
| *Vaccinium vitis-idaea* | 0.1087x + 1.5247 | 0.974 | 7 |

Table S2. ANOVA (F-values) statistics of fatty acid (FA) concentrations in response to the extreme winter warming events (WW) and nitrogen additions (N) for *Empetrum nigrum, Vaccinium vitis-idaea, Pleurozium* *schreberi* and *Cladonia uncialis*. *<0.05, **<0.01. ***<0.001.

| *Empetrum* | 2014 |  |  |  | 2015 |  |  |
| --- | --- | --- | --- | --- | --- | --- | --- |
| FA | WW _(2,26)_ | N _(1,26)_ | WW×N _(2,26)_ | FA | WW _(2,26)_ | N _(1,26)_ | WW×N _(2,26)_ |
|  |  |  |  | C8:0 | 6.4 ** | 1.4 | 1.4 |
| C10:0 | 7.5 ** | 0.8 | 0.3 | C10:0 | 6.4 ** | 1.5 | 1.5 |
| C12:0 | 3.6 * | 0.8 | 2.9 | C12:0 | 1.1 | 6.9 * | 0.1 |
| C13:0 | 4.0 * | 0.5 | 0.8 | C13:1 | 9.0 ** | 0.3 | 2.1 |
| C14:1 | 5.9 ** | 0.2 | 0.1 |  |  |  |  |
| C16:0 | 4.1 * | 3.2 | 0.8 | C16:1 n-7 | 5.9 ** | 1.3 | 1.5 |
| C18:1 n-12 | 1.6 | 5.6 * | 1.7 | C18:0 | 1.7 | 0.0 | 4.7 * |
|  |  |  |  | C18:1 n-7 | 4.5 * | 3.8 | 0.8 |
|  |  |  |  | C18:3 n-3 | 5.9 ** | 1.5 | 1.6 |
| C20:1 n-9 | 4.6 * | 1.0 | 0.4 | C20:2 | 2.9 | 4.1 | 3.7 * |
| C22:1 n-11 | 4.0 * | 0.8 | 0.4 |  |  |  |  |

Table S2 continued

| *Vaccinium* | 2014 |  |  |  | 2015 |  |  |
| --- | --- | --- | --- | --- | --- | --- | --- |
| FA | WW _(2,25)_ | N _(1,25)_ | WW×N _(2,25)_ | FA | WW _(2,25)_ | N _(1,25)_ | WW×N _(2,25)_ |
| C10:0 | 6.9 ** | 1.5 | 1.0 |  |  |  |  |
| C11:0 | 3.4 ** | 1.1 | 1.0 |  |  |  |  |
| C12:1 | 4.1 * | 1.5 | 1.3 | C12:0 | 3.0 ' | 1.6 | 1.2 |
|  |  |  |  | C14:0 | 0.8 | 4.1' | 3.8* |
| C14:1 | 7.5 ** | 1.4 | 0.9 |  |  |  |  |
|  |  |  |  | C16:0 | 2.9' | 1.2 | 1.8 |
| C16:1 n-7 | 7.4 ** | 1.3 | 1.0 | C16:1 n-7 | 0.7 | 3.2' | 0.4 |
|  |  |  |  | C18:2 n-6 | 5.5** | 2.2 | 0.6 |
|  |  |  |  | C21:0 | 3.4* | 4.4* | 1.1 |
|  |  |  |  | C20:2 | 3.3' | 5.2* | 0.9 |

Table S2 continued

| *Pleuroziumm* | 2014 |  |  |  | 2015 |  |  |
| --- | --- | --- | --- | --- | --- | --- | --- |
| FA | WW _(2,26)_ | N _(1,26)_ | WW×N _(2,26)_ | FA | WW _(2,26)_ | N _(1,26)_ | WW×N _(2,26)_ |
|  |  |  |  | C8:0 | 8.8 ** | 2.8 | 2.9 |
|  |  |  |  | C10:0 | 9.0 ** | 3.3 | 3.1 |
|  |  |  |  | C11:0 | 8.9 ** | 3.0 | 2.9 |
|  |  |  |  | C12:0 | 9.3 ** | 3.0 | 3.1 |
|  |  |  |  | C12:1 | 9.6 *** | 2.4 | 3.1 |
|  |  |  |  | C13:0 | 9.3 ** | 2.5 | 2.8 |
|  |  |  |  | C13:1 | 10.2 *** | 3.5 | 2.9 |
|  |  |  |  | C14:1 | 9.5 *** | 2.7 | 3.1 |
| C15:0 | 1.7 | 6.3* | 4.9 * |  |  |  |  |
|  |  |  |  | C16:0 | 1.5 | 3.7 | 5.5 * |
|  |  |  |  | C16:1 n-7 | 9.3 ** | 2.9 | 3.0 |
| C18:1 n-12 | 2.8 | 10.9 ** | 8.9 * | C18:1n-12 | 4.1 * | 0.3 | 0.3 |
|  |  |  |  | C18:2 n-6 | 3.9 * | 3.5 | 6.1 ** |
| C18:3 n-6 | 9.7 *** | 7.8 ** | 5.1 * | C18:3 n-6 | 9.3 ** | 2.6 | 3.1 |
|  |  |  |  | C18:3 n-3 | 0.4 | 4.0 | 3.7 * |
|  |  |  |  | C20:1 n-9 | 9.1 ** | 3.0 | 3.1 |
|  |  |  |  | C20:3 n-6 | 2.7 | 6.4 * | 5.1 * |
|  |  |  |  | C20:5 n-3 | 0.3 | 5.1 * | 2.8 |

Fig. S1. Leaf emergence (%) of *Vaccinium myrtillus, Empetrum nigrum* and *V. vitis-idaea* following extreme winter warming events (WW) and nitrogen additions (N) during three consecutive years. Data points are means of six replicate plot measurements per treatment with SE as error bars. DOY: Day of the year. Statistical analyses results are presented in Table 1.


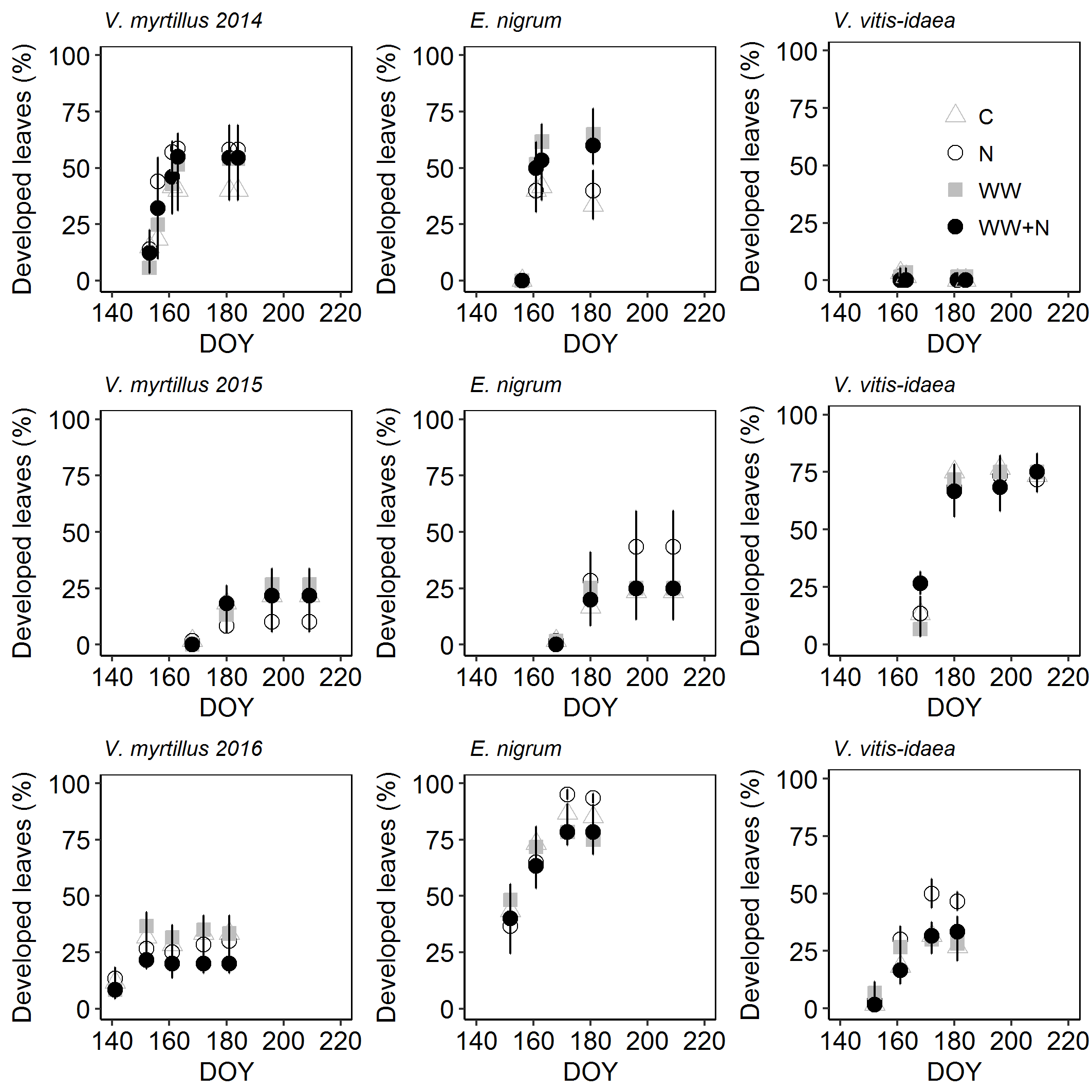


Figure S2. NDVI values following three consecutive (2014-2016) extreme winter warming events (WW) and nitrogen additions (N). Data points are mean of n=6 plots with SE as error bars from recordings during the growing season of 2016. DOY; Day of the year.


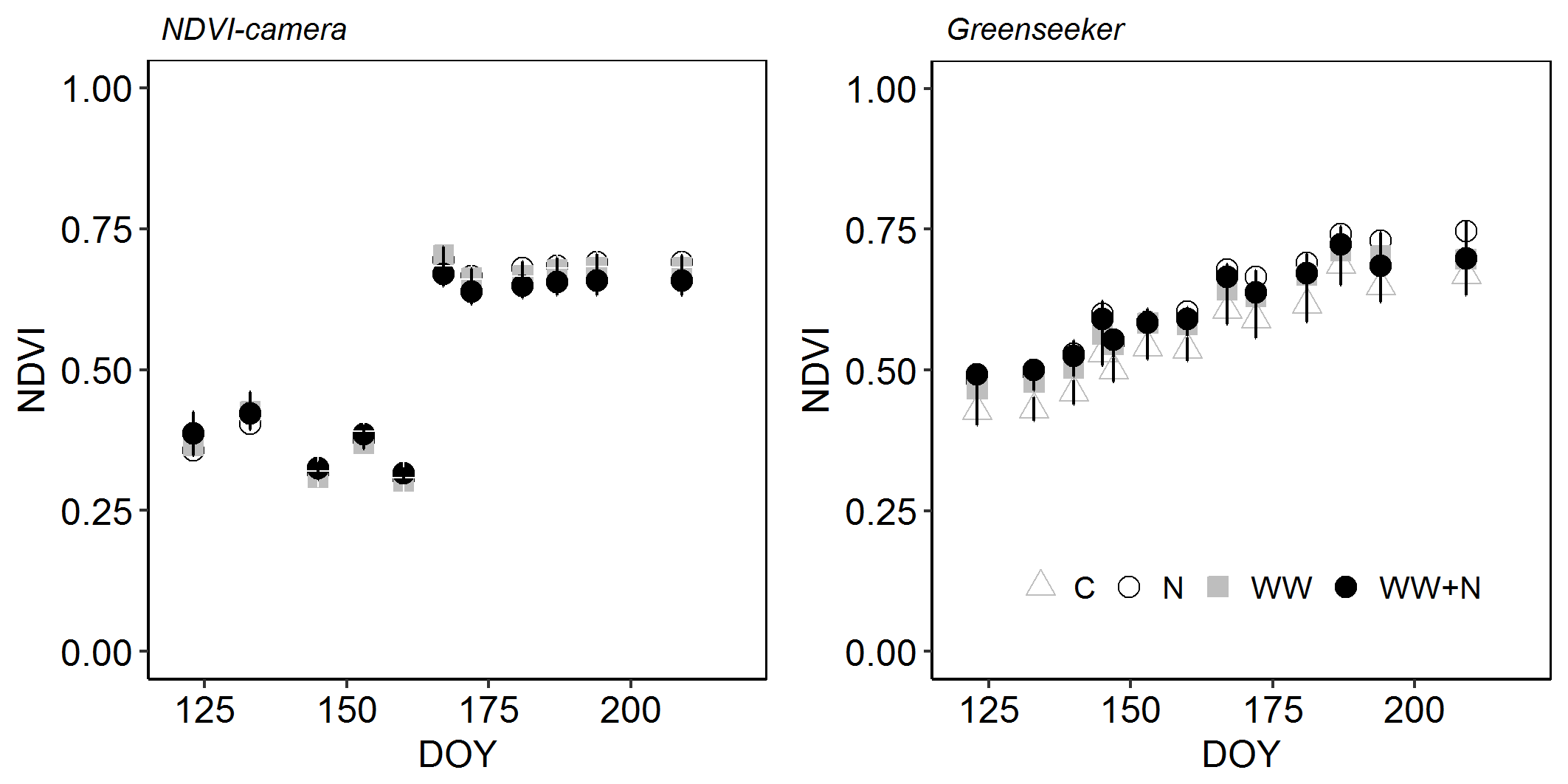


Fig. S3. Growing season ecosystem CO_2_ fluxes during following three consecutive (2014-2016) extreme winter warming events (WW) and nitrogen additions (N). Data points are mean of n=6 plots with SE as error bars. DOY; Day of the year.


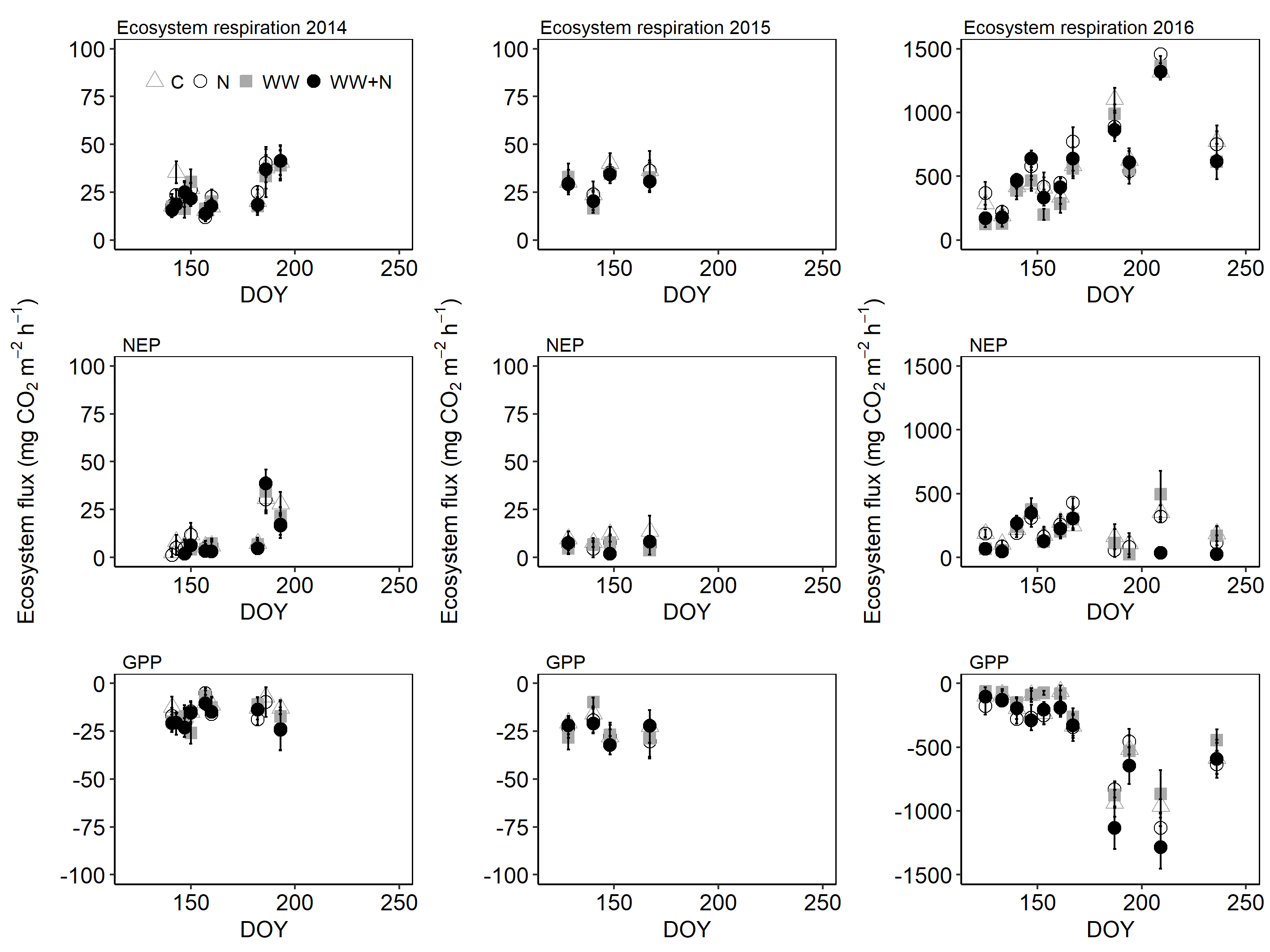

Supplement: Supplementary file 1 [file Table_1.docx]
